# Insights on Localized and Systemic Delivery of Redox-Based Therapeutics

**DOI:** 10.1155/2018/2468457

**Published:** 2018-02-14

**Authors:** Nicholas E. Buglak, Elena V. Batrakova, Roberto Mota, Edward S. M. Bahnson

**Affiliations:** ^1^Department of Surgery, Division of Vascular Surgery, University of North Carolina at Chapel Hill, Chapel Hill, NC 27599, USA; ^2^Center for Nanotechnology in Drug Delivery, University of North Carolina at Chapel Hill, Chapel Hill, NC 27599, USA; ^3^Curriculum in Toxicology, University of North Carolina at Chapel Hill, Chapel Hill, NC 27599, USA; ^4^Division of Pharmacoengineering and Molecular Pharmaceutics, University of North Carolina at Chapel Hill, Chapel Hill, NC 27599, USA; ^5^Department of Cell Biology & Physiology, University of North Carolina at Chapel Hill, Chapel Hill, NC 27599, USA

## Abstract

Reactive oxygen and nitrogen species are indispensable in cellular physiology and signaling. Overproduction of these reactive species or failure to maintain their levels within the physiological range results in cellular redox dysfunction, often termed cellular oxidative stress. Redox dysfunction in turn is at the molecular basis of disease etiology and progression. Accordingly, antioxidant intervention to restore redox homeostasis has been pursued as a therapeutic strategy for cardiovascular disease, cancer, and neurodegenerative disorders among many others. Despite preliminary success in cellular and animal models, redox-based interventions have virtually been ineffective in clinical trials. We propose the fundamental reason for their failure is a flawed delivery approach. Namely, systemic delivery for a geographically local disease limits the effectiveness of the antioxidant. We take a critical look at the literature and evaluate successful and unsuccessful approaches to translation of redox intervention to the clinical arena, including dose, patient selection, and delivery approach. We argue that when interpreting a failed antioxidant-based clinical trial, it is crucial to take into account these variables and importantly, whether the drug had an effect on the redox status. Finally, we propose that local and targeted delivery hold promise to translate redox-based therapies from the bench to the bedside.

## 1. Introduction

Redox reactions are at the center of cellular metabolism and signaling and result in the production of reactive species [1]. Reactive species that stem from oxygen reduction reactions are called reactive oxygen species (ROS) and include free radical and nonradical molecules such as hydroxyl radical (HO^•^), peroxyl (RO_2_^•^), superoxide (O_2_^•−^), singlet oxygen (^1^O_2_), and hydrogen peroxide (H_2_O_2_) [2]. Reactive species that stem from nitric oxide metabolism are called reactive nitrogen species (RNS) and include free radical and nonradical molecules such as nitric oxide (^•^NO) itself, nitrite (NO_2_^−^), nitrogen dioxide (^•^NO_2_), peroxynitrite (ONOO^−^), dinitrogen trioxide (N_2_O_3_), and alky peroxynitrite (ONOOR) [2]. These species are highly reactive and therefore are capable of forming reversible and irreversible interactions with many different macromolecules throughout the body. Under physiological conditions, the reactive species are in balance with antioxidant defenses which enzymatically and nonenzymatically maintain redox homeostasis. In homeostasis, reactive species fulfill their critical role in cell signaling, migration, proliferation, and metabolism. If the redox balance is skewed towards the reactive species however, either from their overproduction or antioxidant depletion, a pathological condition can ensue. Exposure to cigarette smoke [3], various air pollutants [4, 5], UV radiation [6], xenobiotics [7], alcohol consumption [8], and different diseases can further exacerbate the pathological cellular state. Consequently, the prolonged redox imbalance can cause aberrant DNA modifications [9], lipid peroxidation [10], peptide chain fragmentation [11], and alterations in signal transduction [12]. This state of cellular redox imbalance, as ROS and RNS overwhelm defense mechanisms, is often referred to as oxidative stress.

Disruption in redox homeostasis can result in undesirable molecular interactions throughout the cell. Impairment at the molecular level can lead to defective organelles, which can translate into cellular dysfunction. In turn, this can cause tissue incompetence ultimately resulting in the development of organ-specific or systemic disease. For this reason, a component of redox dysfunction has been linked to almost all disease states [13, 14] including asthma, hypertension, carcinoma, leukemia, diabetes, Alzheimer's disease, autoimmunity, and infection. The implication of a causative role of ROS and RNS in disease development has led to many studies and clinical trials using antioxidant molecules as therapeutics. Despite our understanding of the biochemical interaction of reactive species and their associations with disease onset, causality has yet to be fully elucidated. It is because of this poorly understood and missing connection that most redox-based therapies have largely been unsuccessful. For the purposes of this review, we evaluate the research with the assumption that reactive species have a causal role in disease onset. Accordingly, we chose to review a range of redox-based molecules, from micronutrients, which have been trialed for decades, to new and emerging small molecules currently entering clinical trials for the first time. Additionally, we highlight cell-mediated drug delivery as a novel approach for administering these therapeutics. Ultimately, this review aims to rationalize the reasons as to why few redox-based therapies succeed.

## 2. Localized and Targeted versus Systemic Therapy Application

Failure of therapies to date can be attributed to improper route of drug administration, a discrepancy between preventative and remission therapy, wrong dose to achieve desired effect, or the wrong choice in redox-active molecule due to the vast array of reactive moieties between redox-active compounds. We propose that a fundamental issue with most therapeutic approaches up to now has been their method of delivery. For instance, systemic delivery of an antioxidant for the treatment of a geographically local redox imbalance, as with tumors or atherosclerotic lesions, simply may not deliver the therapeutic concentration to the diseased site. The route of administration may not have been fully evaluated or the metabolism of that antioxidant formulation may not have been properly understood before trials began. Moreover, the redox-active therapeutic may interact with and disrupt systems currently at redox homeostasis. This could lead to a state of local reductive or oxidative stress outside of the diseased area as many “antioxidants” have a prooxidant capacity depending on their concentration and redox environment. Following this notion, systemic delivery of antioxidants to treat a disease with systemic redox imbalance, as in diabetes, may yield the desirable effect. For conditions with localized redox imbalance, however, we suggest the necessity of developing locally delivered or targeted therapeutic strategies that deliver the redox intervention directly and preferentially to the site of disease, hence addressing the redox dysfunction in a local and targeted manner.

## 3. Micronutrients

Micronutrients are readily obtained through the diet and were among the first molecules with antioxidant properties to be identified. Their chemical structures have redox-sensitive functional groups or radical scavenging moieties. Additionally, some have the capacity to recycle endogenous antioxidants. These molecules were also the first to be trialed as therapeutics. Herein, we discuss several recent trials with the ubiquitous alpha-lipoic acid as well as vitamins A, B_12_, C, and E.

### 3.1. Vitamin C

Vitamin C, also known as ascorbic acid or ascorbate, is a water-soluble compound synthesized from glucose by most animals and galactose by plants. Humans and apes are among the few animals that lack the key enzyme required for vitamin C biosynthesis, L-gulonolactone oxidase [15]. As such, ascorbate is obtained externally through the diet or by supplementation. Among its many physiological functions, ascorbate acts as a cofactor for neurotransmitter synthesis as well as collagen synthesis and maintenance [16]. As an antioxidant, ascorbate is capable of recycling oxidized vitamin E to its reduced form [17] and scavenging many different ROS and RNS. The preclinical and clinical studies discussed herein are summarized in Table 1.

Work done by Schoenfeld et al. [18] revitalized the therapeutic potential of ascorbate in treating cancer by describing a potential new mechanism in non-small-cell lung cancer (NSCLC) and glioblastoma (GBM). Cellular work using two NSCLC and two GBM cell lines showed that ascorbate selectively sensitizes these cancer cells to chemotherapeutics and radiation therapy compared to primary bronchial epithelial cells and normal human astrocytes [18]. As previously mentioned, antioxidants can have a prooxidant capacity depending on their redox environment. Here, ascorbate treatment increased steady-state levels of hydrogen peroxide (H_2_O_2_) and labile iron levels in both cancer cell types, but not in noncancerous cells [18]. The enhanced cancer cell-ascorbate toxicity could be inhibited by preincubation with iron chelators or with catalase, which breaks down H_2_O_2_. Meanwhile, preincubation with EDTA [18], a known enhancer of iron redox cycling, further increased cancer cell-specific toxicity. Lane et al. [19] reported that ascorbate may also increase intracellular iron uptake by acting on transferrin receptors. The cancer-specific toxicity *in vitro* was recapitulated in mouse xenograft models by Schoenfeld et al. [18] for both NSCLC and GBM using combinations of radiochemotherapy with intraperitoneal injections of ascorbate. Animals treated with all three therapies, compared to radiochemotherapy or ascorbate alone, exhibited an increased rate of survival [18]. The authors proposed a mechanism inherent to cancer cells whereby superoxide (O_2_^•−^) and H_2_O_2_ disrupt cellular iron metabolism with ascorbate oxidation further increasing intracellular H_2_O_2_ and labile iron levels. Through the Fenton reaction, H_2_O_2_ and iron react causing an increased oxidative burden and resulting in the observed cancer-specific ascorbate toxicity [18]. A phase I clinical trial for GBM patients treated with radiation, temozolomide, and intravenous ascorbate infusions showed that ascorbate was well tolerated and increased the average progression-free survival compared to the historical median [18]. Additionally, a phase II clinical trial for NSCLC patients treated with chemotherapy and ascorbate infusions reported an increased disease control rate compared with historical controls [18]. Unfortunately, the efficacy of ascorbate infusions in concert with radiochemotherapy has yet to be determined due to the limited sample size in these two studies.

The ability of ascorbate to specifically target NSCLC and GBM cancer cells without affecting the redox homeostasis of healthy cells makes systemic intravenous administration possible. In this example, the redox modulation happens only in tumor cells where iron metabolism is disrupted, and in this sense, this is an example of targeted therapy. However, a consistent plasma concentration must be maintained and 20 mM has been proposed as the therapeutic optimum [20]. Meanwhile, oral administration is incapable of obtaining and maintaining the required plasma concentrations [21, 22]. These data show the importance of administration route to achieve the desired effect. In the PACMAN study, a phase I clinical trial for stage IV pancreatic cancer, Welsh et al. [23] showed that infusion of high doses of ascorbate together with gemcitabine achieved stable plasma levels around 20 mM. The PACMAN study also revealed that systemically administered ascorbate did not cause an alteration in the erythrocyte reduced to oxidized glutathione (GSH:GSSG) ratio nor in plasma levels of F_2_-isoprostane [23], an arachidonic acid peroxidation product commonly used as a biomarker. The mean survival was 12 months, almost double the historic median for gemcitabine-treated patients [23], yet due to the small sample size, no statistically significant conclusion about efficacy can be made. As of June 2017, the PACMAN study is scheduled to enter phase II (NCT02905578), with phase II trials for NSCLC (NCT02905591) and GBM (NCT02344355) scheduled as well. Hopefully, these upcoming and future trials will identify if an intravenous vitamin C formulation is an effective targeted systemic delivery for treating a geographically local redox imbalance.

### 3.2. Vitamin E

E vitamins are a group of fat-soluble compounds classified as saturated tocopherols or unsaturated tocotrienols, with *α*, *β*, *γ*, and *δ* isoforms within each group. The most biologically active vitamin E compound in humans is *α*-tocopherol. Therefore, *α*-tocopherol will be the vitamin E isoform described henceforth unless otherwise specified. Interestingly, vitamin E has been hypothesized to act only as a peroxyl (RO_2_^•^) radical scavenger [17, 24]. As such, vitamin E effectively prevents the peroxyl radical chain reaction-driven lipid peroxidation. A summary of the preclinical and clinical studies discussed can be seen in Table 2.

Being fat soluble, vitamin E is mainly transported by lipoproteins where it prevents lipoprotein oxidation [25]—a redox reaction that drives atherosclerotic plaque development. An atherosclerosis model of rabbits fed a high-cholesterol diet showed that dietary vitamin E supplementation significantly reduced plaque development [26]. Tang et al. showed that vitamin E supplementation reduced plaque development in apolipoprotein E knockout (ApoE^−/−^) mice when administered early, between 6 and 22 weeks of age, yet had no effect on reducing advanced lesions when administered at 30 or 38 weeks [27]. It is worth noting here that *α*-tocopherol has been shown to have limited antiatherosclerotic potential in rodent models [28]. In both the rabbit model and the ApoE^−/−^ mice, vitamin E activated the nuclear factor erythroid 2-related factor 2 (Nrf2) pathway [26, 27], which regulates the expression of antioxidant enzymes [29]. Specifically, Bozaykut et al. [26] showed an increase in the expression of glutathione S-transferase, one downstream enzyme of Nrf2, which is responsible for initiating the function of glutathione (GSH) [30], another major intracellular antioxidant. The Nrf2 pathway has now also been implicated as a vitamin E therapeutic mechanism in *in vitro* models of cancer [31] and macular degeneration [32] as well as in *in vivo* allergy [33] and developmental [34] studies. Furthermore, vitamin E has also been shown to increase peroxisome proliferator-activated receptor gamma (PPAR*γ*) expression [26, 27]. PPAR*γ* is one isoform of the PPAR ligand-activated transcription factors whose activation can be protective against cardiovascular diseases, neurodegenerative disorders, and cancer [35–37]. Thus, Nrf2 and PPAR activation reveal indirect antioxidant properties of vitamin E, which when paired with the direct radical scavenging ability make for a promising therapeutic.

CHAOS [38] and HOPE [39] were two of the first trials using vitamin E in prevention of cardiovascular disease. Both of these trials showed no cardiovascular benefit, which can potentially be attributed to the 400 IU/day [39] or up to 800 IU/day [38] doses used. In 2007, Roberts et al. [40] conducted both a dose-ranging and time-course study in hypercholesterolemic patients to evaluate the pharmacology of vitamin E. This was a beneficial study for future clinical trials as they revealed two critical dosing aspects. First, it took 16 weeks of daily dosing to reduce the plasma F_2_-isoprostane concentration [40]. Second, this reduction was only significantly obtained at 1600 IU/day and 3200 IU/day, with a trend towards reduction at 800 IU/day [40]. At the largest dose of natural vitamin E (3200 IU/day), there was a 49% reduction in plasma F_2_-isoprostanes, which led the authors to suggest that the antioxidant potency of vitamin E may not be biologically effective [40]. These studies taken together underline the relevance of selecting the appropriate dose and regimen to achieve a significant effect on redox homeostasis. Only after determining that a treatment actually improves redox function can we assess its antioxidant effect on disease progression and clinical outcomes. A study by Milman et al. [41] showed that daily vitamin E supplementation only had cardiovascular benefit in type 2 diabetics having a haptoglobin 2-2 genotype. The protein produced by this genotype is associated with elevated systemic oxidative stress due to its inferior function compared to the haptoglobin 1-1 genotype protein product. Several studies measured serum malondialdehyde (MDA) levels, a lipid peroxidation product, to assess the benefit of oral vitamin E supplementation. Although a reduction in plasma MDA levels was frequently observed, this systemic decrease in a lipid peroxidation marker did not correlate with improvement of clinical symptoms [42–44]. Thus, translating cellular and animal model work into an effective human therapeutic while also identifying the proper patient population that benefits from the given therapy and relevant biomarkers makes clinical trial design rather difficult.

Patients with ranging neurological disorders have been reported to experience increased levels of systemic oxidative stress [13]. Parisotto et al. [45] compared the blood antioxidant status of children with Down syndrome (DS) before and after 6 months of daily vitamin C and E supplementation. The study showed that DS patients had elevated superoxide dismutase (SOD) and catalase activity yet lower GSH levels, and all could be returned to physiological levels after supplementation [45]. A randomized, double-blind, placebo-controlled, parallel-group trial reported that a high dose of 2000 IU/day of vitamin E slowed the functional decline of patients with mild to moderate Alzheimer's disease [46]. Both studies highlight how a systemically administered antioxidant can reduce systemic redox imbalance and alleviate symptoms experienced by individuals with neurological disease.

Lastly, Alias et al. [47] showed that subcutaneous application of vitamin E acetate ointment reduced the rate of surgical site infection in colorectal cancer surgery patients, while simultaneously lowering the inflammatory response. The patients underwent a laparoscopic, or minimally invasive, surgery. Patients who received vitamin E treatment had decreased C-reactive protein and a reduced white blood cell count 48 hours after surgery and reported lower postoperative pain [47]. This is an excellent example of a preventative strategy for localized delivery of an antioxidant to mitigate the onset of local redox imbalance.

### 3.3. Alpha-Lipoic Acid

The disulfide fatty acid *α*-lipoic acid (ALA) is an amphipathic molecule both naturally synthesized and obtained through the diet. Virtually ubiquitous throughout the body, ALA and its reduced form, dihydrolipoic acid, have been reported to have numerous antioxidant and anti-inflammatory properties [48]. The therapeutic potential thus stems from the capability of ALA to scavenge reactive species [49, 50], recycle glutathione (GSH) and vitamins C and E [49], chelate divalent transient metal ions [50], and increase glucose uptake [51]. The preclinical and clinical studies described from here on are summarized in Table 3.

Goraca et al. [52] evaluated ALA treatment in a rat model of bacterial infection using lipopolysaccharide (LPS), a major component of the outer membrane of gram-negative bacteria. The spleen of LPS-treated rats had increased levels of hydrogen peroxide (H_2_O_2_) and lipid peroxidation, an increased spleen weight/body weight ratio, and decreased levels of GSH [52]. All three of these markers were returned to physiological levels after intravenous ALA injection [52] suggesting that ALA can protect the spleen from an exaggerated inflammatory response. Cimolai et al. [53] showed that intraperitoneal-injected ALA could restore kidney mitochondrial function in rats exposed to LPS. Mignini et al. [54] used a pesticide-induced oxidative stress rat model to evaluate the effects of oral ALA supplementation and showed that ALA could increase plasma GSH concentrations. Additionally, ALA restored SOD and catalase plasma concentrations to physiological levels [54]. Subcutaneous delivery of ALA restored SOD and catalase to physiological levels in the brain tissue of a phenylketonuria rat model [55], a neurological disease that causes systemic phenylalanine accumulation and oxidative stress. Orally administered ALA slowed the rate of plaque progression, lowered plasma F_2_-isoprostane levels, decreased lipid accumulation, and reduced collagen deposition in a diet-induced rabbit atherosclerosis model [56]. Furthermore, ALA decreased mRNA expression of vascular cell adhesion molecule-1 (VCAM-1) and of intracellular adhesion molecule-1 (ICAM-1) and reduced NF-*κ*B pathway activity in the rabbit model [56]; all of which are proinflammatory factors associated with plaque development [57, 58]. A diet-induced atherosclerotic mouse model also showed that intraperitoneal ALA delivery could reduce lesion size and VCAM-1 and ICAM-1 expression [59]. Lee et al. [59] showed that ALA could reduce vascular smooth muscle proliferation and migration *in vitro* by attenuating the Ras-MEK1/2-ERK1/2 proliferative pathway. As of June 2017, phase III clinical trials using ALA for atherosclerosis treatment (NCT00764270) and heart disease prevention (NCT00765310) are ongoing.

ALA supplementation has been particularly successful in diabetic patients and has been prescribed for the treatment of diabetic polyneuropathy in Germany for decades [50]. In 2004, Ziegler et al. [60] assessed the role of oxidative stress in the development of diabetic polyneuropathy, a complex neuronal disorder associated with increased morbidity and mortality. Their results showed that systemic redox imbalance was more prominent in diabetics with polyneuropathy than in diabetics without polyneuropathy [60]. The SYDNEY 2 clinical trial assessed the efficacy of orally administered ALA in alleviating pain experienced by diabetic patients with distal symmetric polyneuropathy [61]. The primary measurement in the SYDNEY 2 trial was total symptom score (TSS), a summation of factors related to neuropathic pain where higher scores indicate an increased level of experienced pain. The results revealed that 600 mg of ALA administered orally for 5 weeks improved the positive sensory symptoms of diabetic patients [61]. Similar findings were described by Garcia-Alcala et al., [62] who reported that 20 weeks of ALA treatment resulted in a continuous reduction in TSS. NATHAN 1 was a four-year clinical trial that further assessed ALA treatment for polyneuropathy as well as the safety of prolonged oral administration of 600 mg of ALA [63]. This trial used several comprehensive analyses alongside TSS such as the neuropathy impairment score (NIS) [64] and the NIS lower limbs score to measure therapeutic outcomes without any direct measurements of changes in redox markers. Improvement of neuropathic impairments, particularly small fiber and muscular function, was ultimately reported, and the 600 mg dose was well tolerated by patients [63]. We guide the readers to an extensive review by Papanas and Ziegler [65] of more trials and relevant meta-analyses regarding ALA in diabetic neuropathy. The success seen in these trials can likely be attributed to the hydrophilic and lipophilic nature of ALA as well as to the delivery route in treating a disorder that stems from a systemic redox imbalance.

### 3.4. Vitamin A

A vitamins are a group of unsaturated, fat-soluble organic molecules that elicit pleiotropic effects throughout the body. Physiologically, vitamin A regulates energy homeostasis, lipid metabolism, the immune response, and gene expression for cellular development and differentiation [66–68]. The major signaling mechanism for the regulatory actions of vitamin A is through nuclear retinoic acid receptors. However, signaling through Janus kinase (JAK)/signal transducer and activator of transcription (STAT), mitogen-activated protein kinase (MAPK), and PPAR pathways have also been described [67]. Vitamin A quenches singlet oxygen (^1^O_2_) [69] and prevents lipid peroxidation by scavenging peroxyl (RO_2_^•^) radicals [70], similar to vitamin E. Furthermore, the major active metabolite of vitamin A—retinoic acid—acts as a precursor for rhodopsin, the light-sensitive pigment in the eye responsible for phototransduction [71]. Retinoic acid is obtained through the diet from animal-derived food or from plant-derived precursors like *β*-carotene and lycopene. Clinical studies using vitamin A are summarized in Table 4.

In accordance with the pleiotropic properties on cellular homeostasis, vitamin A was trialed and is now accepted as a therapeutic for various skin-related conditions. Topically applied *β*-carotene protected human skin from IR-generated reactive species [72] and orally administered *β*-carotene reduced UV-induced skin lesions [73]. A randomized, double-blind, vehicle-controlled, clinical trial showed that topical retinol, the alcohol retinoic acid derivative, applied three times a week for 24 weeks reduced the appearance of aging-related fine wrinkles in elderly volunteers by increasing the dermal matrix [74]. Furthermore, topical application of tretinoin or all-*trans* retinoic acid has long been prescribed for the treatment of acne and photoaging [75, 76] and is more effective than orally administered tretinoin in cases of photoaging [77]. The role of these and more vitamin A derivatives in treating skin disease has been reviewed elsewhere [78]. The success of topical vitamin A application provides evidence that identifying the proper antioxidant for a given condition combined with an administration route that localizes the redox intervention results in a successful antioxidant therapy.

### 3.5. Vitamin B_12_

Vitamin B_12_ derivatives or cobalamins (Cbl) are soluble macrocycles belonging to the corrinoid family with a cobalt atom tethered in the center of the corrin plane. They are essential cofactors for two enzymes in mammals: mitochondrial methylmalonyl-CoA mutase and cytosolic methionine synthase. The central cobalt can change its oxidation state between +3 (cob(III)alamin), +2 (cob(II)alamin), and +1(cob(I)alamin), which makes these compounds redox active. Moreover, it has been shown that Cbl reacts with O_2_^•−^ at rates comparable with that of superoxide dismutase [79, 80]. Hence, it has been suggested that cobalamins modulate inflammation and redox status [81–83] in addition to and independently of their cofactor function. Low serum levels of B_12_ are prevalent in patients with type 2 diabetes mellitus, and there is an association between low levels of B_12_ and markers of oxidative dysfunction [84]. Moreover, levels of B_12_ negatively correlate with fasting glucose, oxidized LDL, and catalase levels in diabetic vegetarians [85]. These findings suggest that Cbl supplementation could be useful in patients with type 2 diabetes mellitus, especially if they are vegetarians, who are at risk of low B_12_. Preclinical and clinical work with vitamin B_12_ is summarized in Table 5.

Cobalamin is essential to metabolize homocysteine, an independent risk factor for cardiovascular disease. Hence, researchers have investigated the role of Cbl in cardiovascular disease. One of us was among the first to characterize a redox effect in vascular cells [83] and suggest that it acted by direct scavenging of O_2_^•−^ with a high rate constant [80]. In a randomized placebo-controlled trial, a mix of folate, vitamin B_6_, and vitamin B_12_ reduced the carotid intima-media thickness in patients at risk of cerebral ischemia [86]. This effect was found to be independent of homocysteine. In a small prospective randomized placebo-controlled trial, Willems et al. [87] demonstrated that treatment with cobalamin in patients with coronary artery disease improved volumetric coronary blood flow. These results were later confirmed by Bleie et al. [88] showing an increased coronary blood flow in a patient with stable coronary artery disease treated with cobalamin, suggesting that B_12_ improves vascular function.

Cobalamin levels are associated with systemic and central nervous system (CNS) markers of redox dysfunction and inflammation, independently of homocysteine levels [82]. In a controlled, double-blinded study in patients with idiopathic osteoarthritis, Flynn et al. [81] found that Cbl improved handgrip and tenderness compared to NSAID treatment. Regarding effect on the CNS, intramuscular injection of high doses of Cbl improved muscle action potential amplitude in a small double-blind trial in patients with amyotrophic lateral sclerosis [89].

A successful example of local administration of Cbl to reduce redox dysfunction and inflammation was published by Macri et al. [90]. They found that eye drops containing hyaluronic acid and vitamin B_12_ significantly decrease markers of lipid peroxidation and inflammation, improving dry eye symptoms. Although vitamin B_12_ is not classically considered an antioxidant, more evidence accumulates supporting a redox effect of Cbl. Since it is well tolerated and no adverse effects have been reported, exploring further the noncoenzyme and redox effects of Cbl is a promising therapeutic avenue for cardiovascular, neurological, and inflammatory diseases.

## 4. Enzyme Regulators and Mimetics

**Figure 1:**
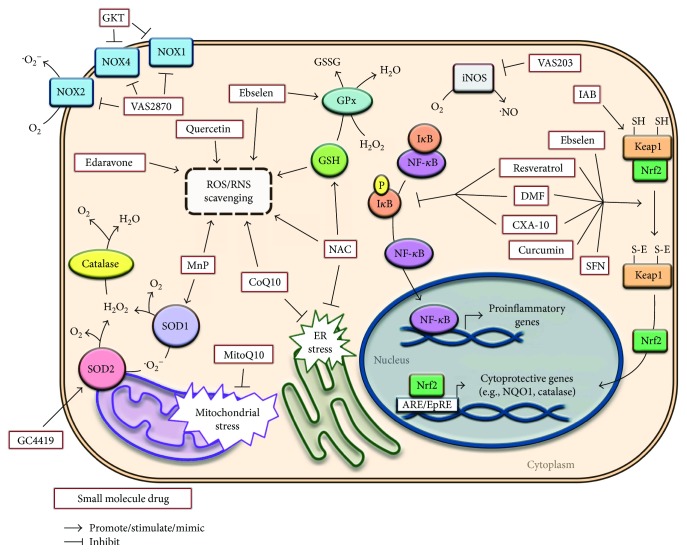
A scheme depicting cellular sites of action of several redox interventions. ARE/EpRE: antioxidant response element/electrophile responsive element; CoQ10: coenzyme Q10; CXA-10: 10-nitro-oleic-acid formulation; DMF: dimethyl fumarate; ER: endoplasmic reticulum; GKT: GKT137831; GPx: glutathione peroxidase; GSH: glutathione; IAB: N-iodoacetyl-N-biotinylhexylenediamine; MnP: manganese porphyrins; NAC: N-acetylcysteine; NOS: nitric oxide synthase; NOX: NADPH oxidase; NQO1: NAD(P)H quinone dehydrogenase 1; Nrf2: nuclear factor erythroid 2-related factor 2; RNS: reactive nitrogen species; ROS: reactive oxygen species; SFN: sulforaphane; SOD: superoxide dismutase.

The use of stoichiometric antioxidant molecules presents with the problem that they are finite. At the necessary site they may be present in limited concentrations or may not have been recycled after their redox reaction. Accordingly, most cellular redox homeostasis is maintained by enzymes [91]. As we have shown with micronutrients, certain antioxidants can increase enzyme production and function aside from directly inactivating the reactive species. Herein, we analyze several specific enzyme-directed therapeutics and present an overview in Figure 1. Next, we discuss the use of redox enzymes, enzyme mimics, and enzyme regulators.

### 4.1. NADPH Oxidase Inhibitors

Nicotinamide adenine dinucleotide phosphate (NADPH) oxidases (NOX) are a group of transmembrane enzymes that generate superoxide (O_2_^•−^) by transferring electrons from NADPH across a membrane to reduce molecular oxygen [92]. Accordingly, NOX enzymes are present in the plasma membrane, endoplasmic reticulum, mitochondrial membrane, and nuclear membrane, as well as at focal adhesions [93] and invadopodia [94]. In total, there are seven NOX isoforms termed NOX1-5 or DUOX1-2. NOX enzymes are physiologically crucial for the immune response and during angiogenesis; these functions along with enzymatic regulation and localization have been reviewed in extensive detail elsewhere [95]. Pathologically, dysregulated NOX enzymes have been implicated in various cancers [96, 97], neurodegenerative diseases [98], cardiovascular diseases [99], and renal disease [100].

Due to the association of dysregulated NOX with disease onset, it was fitting to pursue direct NOX inhibition as a therapeutic. Triazolopyrimidine derivatives (VAS) and pyrazolopyridine dione derivatives (GKT) are two efficacious small molecule inhibitors that have been reported. VAS and GKT successfully mitigate ROS formation and damage in *in vitro* and *in vivo* models of disease [101–105]. These two families, and others not covered in this section, have been extensively reviewed elsewhere [106].

Specifically, GKT137831 is a NOX 1 and 4 inhibitor that has been reported to mitigate renal disease [105], attenuate diabetic nephropathy [102], and suppress cardiac fibroblast activity associated with hypertensive heart disease [104]. A randomized, double-blind, placebo-controlled phase II trial using GKT137831 for treating type 2 diabetes with nephropathy was concluded in 2015 (NCT02010242). The drug developer, Genkyotex, reported that GKT137831 was safe and significantly reduced liver enzyme and inflammatory marker levels, with the results unpublished as of June 2017. Currently, Genkyotex is finalizing a phase II study design to treat the liver disease primary biliary cholangitis with GKT137831.

Meanwhile, VAS2870 a NOX 1, NOX 2, and NOX 4 inhibitor has been reported to have protective properties against brain ischemia in mice [101] and to reduce ROS production and radiation-induced phenotypic changes in human pulmonary artery endothelial cells [103]. One challenge for translating the success of VAS molecules to the clinic is their low solubility. Hecht et al. [107] have potentially solved this issue by developing a method that utilizes spray drying, microemulsification, and cyclodextrin incorporation to enhance the solubility and stability of VAS3947 as a model VAS molecule, for oral administration. Ongoing studies with these two classes of molecules will offer a greater understanding of their pharmacological efficacy and the value of targeted NOX inhibition in disease treatment or prevention.

### 4.2. Nitric Oxide Synthase Inhibitors

Nitric oxide synthase (NOS) represents a group of enzymes that utilize molecular oxygen and NADPH to oxidize L-arginine producing L-citrulline and nitric oxide (^•^NO). There are three NOS isoforms termed neuronal NOS (nNOS or NOS I), inducible NOS (iNOS or NOS II), and endothelial NOS (eNOS or NOS III). Exceptions to the general locations and regulatory mechanisms for each isoform have been extensively reviewed elsewhere [108], but generally, nNOS is found throughout the central (CNS) and peripheral (PNS) nervous systems where ^•^NO is implicated in aspects of synaptic plasticity and cellular communication; iNOS is typically found in cells of the immune system where ^•^NO is responsible for the cell's cytotoxic properties; eNOS is mostly expressed in endothelial cells where ^•^NO causes vasodilation and vasoprotection and prevents platelet aggregation. Therapeutic approaches have been aimed at inhibiting iNOS.

Initially, NOS inhibitors aimed to displace the enzymatic substrate arginine but were clinically ineffective and even increased mortality in the case of L-NMMA treatment for septic shock [109]. To date, the most successful NOS inhibitor is 4-amino-tetrahydrobiopterin (VAS203), developed by Vasopharm, which is an analogue of the redox-sensitive NOS cofactor tetrahydrobiopterin rather than an arginine analogue [110]. VAS203 is an allosteric iNOS inhibitor and is being trialed for the treatment of traumatic brain injury (TBI). TBI has been shown to cause increased iNOS activity in the brain due to a compromised blood-brain barrier, aside from other major complications [111]. An exploratory phase II placebo-controlled, randomized trial named NOSTRA-II determined that VAS203 may have neuroprotective properties and can be safely administered, although there was reversible kidney damage reported in several patients at the highest tested dose (30 mg/kg) [110]. In light of these findings, a phase I trial to assess renal function impairment in healthy volunteers was concluded in early 2017 with results unreported so far (NCT02992236). As of June 2017, NOSTRA-III—the placebo-controlled, randomized, double-blind phase III trial—is underway to evaluate the efficacy of VAS203 infusions on clinical outcomes of patients with moderate to severe TBI (NCT02794168).

### 4.3. Superoxide Dismutase Mimetics

Superoxide dismutase (SOD) is a group of metal-containing enzymes that convert superoxide (O_2_^•−^) to hydrogen peroxide (H_2_O_2_) and/or molecular oxygen. In humans, there are cytosolic (SOD1) and extracellular (SOD3) isoforms which utilize copper and zinc as cofactors while the mitochondrial (SOD2) isoform uses manganese as the cofactor. Manganese porphyrins (MnP) are among the most prominent SOD mimics developed to date. MnPs are made up of a macrocyclic ring structure with manganese found at the center. Other SOD mimics also have similar structures but house a different metal at their center. Further detail of MnP alongside other prominent mimetics' synthesis, structure, kinetics, and potential therapeutic benefits has been extensively reviewed [112]. The therapeutic efficacy of MnPs is heavily dependent on the redox environment at the time of administration. For instance, MnP administration at the onset of diabetes inhibited oxidative damage in a rat model [113]. When administered after the onset of diabetes, however, the same MnP actually increased oxidative damage [114].

GC4419 is a promising new MnP-like SOD mimetic highly specific for reacting with O_2_^•−^. GC4419 has been shown to rescue cell clonogenicity of both a SOD2-*null* human embryonic kidney cell line [115] and radiation or chemotherapy-treated primary human dermal fibroblasts [116]. Intravenous delivery of GC4419 is currently in a phase II clinical trial for alleviating radiation-induced oral mucositis in patients with head and neck cancer (NCT02508389). Galera Therapeutics Inc. is also assessing the tolerability and pharmacokinetics of another SOD mimetic, GC4711, given orally compared to intravenous GC4419 in healthy volunteers (NCT03099824). BMX-001 is a MnP currently in a phase I clinical trial for glioblastoma patients undergoing radiation therapy (NCT02655601). Additionally, a continuous infusion of MnP AEOL 10150 using a subcutaneously implanted osmotic pump was able to significantly reduce radiation-induced injury in a rat model [117] and has proven to be beneficial in a nonhuman primate radiation-induced injury model [118]. As of 2017, Aeolus Pharmaceuticals Inc. is working on moving AEOL 10150 into clinical trials. Of note, MnPs often can react with various species and moieties besides O_2_^•−^ which expands their potential outside of being just a SOD mimetic but also complicates their utility.

### 4.4. Catalase Delivery

Catalase is an antioxidant enzyme that catalyzes the disproportionation of H_2_O_2_ to O_2_ and H_2_O; it decreases neuroinflammation and attenuates neurodegeneration in the CNS.

An important component of many metabolic and inflammatory diseases of the CNS involves inflammation [119]. These include Alzheimer's and Parkinson's diseases (AD and PD) [120–122], stroke [123, 124], traumatic brain injury (TBI), multiple sclerosis (MS), age-related macular degeneration (AMD), prion disease, meningitis, encephalitis, human immunodeficiency virus- (HIV-) associated neurocognitive disorders [125, 126], epilepsy, brain cancer, lysosomal storage diseases [127, 128], obesity [129, 130], and even mental health disorders such as depression, autism, and schizophrenia. These diseases are often accompanied by the extensive recruitment of immunocytes. Such inflammatory activities are linked to microglial activation and its secretion of neurotoxic factors. These include ROS and RNS leading to oxidative stress and neuronal injuries [131–134]. Oxidative stress affects neuronal, astrocyte, and microglia function by inducing ion transport and calcium mobilization and activating apoptotic programs. Apoptosis and excitotoxicity are principal causes of mitochondrial-induced neuronal death. Therefore, the need for delivery of potent antioxidants, in particular, catalase to affected brain tissues cannot be overstated.

The administration of catalase has been shown to rescue the primary cultured cerebellar granule cells in *in vitro* models of PD [135, 136]. Thus, addition of catalase to cerebellar granule cells treated with a toxin, 1-methyl-4-phenyl-1,2,3,6-tetrahydropyridine (MPTP) that causes a severe and irreversible parkinsonian syndrome in humans and in nonhuman primates [137], resulted in enhancing their viability by 75% compared to the control cells. Regrettably, the blood-brain barrier (BBB) severely restricts the transport of this potent antioxidant to the brain. Although small lipophilic molecules (MW < 400 kDa) can cross the BBB in pharmacologically significant amounts, effective concentrations of lipid-insoluble drugs (polar molecules and small ions), as well as high molecular weight compounds (peptides, proteins, and nucleic acids), cannot be delivered to the CNS within the limits of clinical toxicity. The inability of most potent therapeutics to cross the BBB following systemic administration necessitates the need to develop unconventional, clinically applicable drug delivery systems for the treatment of brain disorders. Smart, biologically active vehicles are crucial to accomplishing this challenging task.

To this end, immunocytes that include mononuclear phagocytes (dendritic cells, monocytes, and macrophages), neutrophils, and lymphocytes can carry nanoformulated drugs as they readily home to disease sites. Using living cells as drug delivery vehicles, takes advantage of their natural carriage, storage, mobility, and secretory capacity. It offers several benefits over common drug administration regimens; these include targeted drug transport to disease sites, prolonged drug half-lives, time-controlled drug release, and diminished drug immunogenicity and cytotoxicity profiles. Thus, a cell-based delivery system to bring nanoformulated catalase to affected brain regions was developed [138, 139]. The system rests in the abilities of blood-borne macrophages to carry antioxidant proteins across the BBB to the inflamed tissues. Using inflammatory response cells enables targeted drug transport and prolonged circulation times [140], along with reductions in cell and tissue toxicities. In addition, these cells are capable of cell-to-cell transmission of drug-laden nanoparticles that improves their therapeutic outcomes.

One of the main obstacles in utilizing these cells is related to the fact that monocytes efficiently disintegrate and digest all entrapped foreign particles. Therefore, it is crucial to protect the activity of the drug inside this cell carrier [141]. To preclude macrophage-mediated enzyme degradation, catalase was packaged into a block ionomer complex with a cationic block copolymer, poly(ethyleneimine)-poly(ethylene glycol) (PEI-PEG). Overall, the structure and composition of protective nanocontainers play a crucial role in the effectiveness of formulations for cell-based drug delivery systems. For example, a recent study by one of us indicates that structure of block copolymer used for catalase nanoformulation (nanozyme), affects its cytotoxicity, loading, and release capacities, as well as preservation of catalase enzymatic activity inside cell-carriers [142]. Bone marrow-derived macrophages (BMM) carried significant amounts of catalase then slowly released the active enzyme over several days [138]. The enzyme released upon stimulation of nanozyme-loaded cell carriers decomposed microglial H_2_O_2_ produced upon nitrated alpha-synuclein or tumor necrosis factor alpha- (TNF-*α*-) induced activation *in vitro*. A significant amount of catalase was detected in brains of mice after transfer of BMM loaded with nanoformulated catalase following MPTP intoxication. It was demonstrated that such nanozyme-loaded BMM injected into MPTP-intoxicated mice reduce neuroinflammation and attenuate nigrostriatal degeneration [139]. This signified a neuroprotective effect of nanozyme-loaded monocytes in MPTP-induced neurodegeneration.

Another approach to targeted cell-mediated brain delivery of catalase is to utilize macrophages transfected *ex vivo* with a plasmid DNA (pDNA) encoding catalase [143]. Systemic administration of pretransfected macrophages produced month-long expression levels of catalase in the brain resulting in threefold reductions in inflammation and complete neuroprotection in mouse models of PD. This resulted in significant improvements in motor functions in PD mice. Mechanistic studies revealed that transfected macrophages secreted extracellular vesicles, exosomes, packed with catalase genetic material, pDNA and mRNA, active catalase, and NF-*κ*B, a transcription factor involved in the encoded gene expression. Exosomes efficiently transfer their contents to contiguous neurons resulting in de novo protein synthesis in target cells. Thus, genetically modified macrophages serve as a highly efficient system for reproduction, packaging, and targeted gene and drug delivery to treat inflammatory and neurodegenerative disorders [143]. Of note, a proper differentiation of drug carriers into particular subtypes may further boost the therapeutic efficiency of cell-based drug formulations [144]. Alternatively, activated macrophages phenotype (M2) utilized in these studies did not promote further inflammation in the brain, resulting in a subtle, but statistically significant effect on neuronal regeneration and repair *in vivo*.

Even though this modality of antioxidant enzyme delivery has not moved to clinical trials yet, it is a promising approach to deliver redox-active enzymes in a targeted manner and to overcome several of the obstacles related to delivery of proteins and delivery to the CNS.

## 5. Small Molecule Antioxidants

Small (MW <250 kDA) molecule antioxidants have desirable pharmacokinetic properties able to exponentially enhance antioxidant defense by directly acting on signaling pathways [145]. Phenolic groups dominate this class but there are multiple other species with unique antioxidant properties [146]. In this section, we will discuss some of the most commonly used and already developed small molecule antioxidants and highlight emerging formulations (Figure 1).

### 5.1. Electrophiles

Electrophilic small molecules are potent activators of the nuclear factor erythroid 2-related factor 2 (Nrf2) pathway. Nrf2 is a transcription factor that induces the production of antioxidant and detoxification enzymes controlled by an enhancer sequence called the antioxidant response element/electrophile responsive element (ARE/EpRE). The identification of Nrf2 as the critical transcription factor in this pathway was only recognized in 1997 [29]. This discovery triggered tremendous progress in understanding the increased efficacy of small molecule antioxidants in therapeutic applications. Under homeostatic conditions, Nrf2 is sequestered to the cytoplasm by the actin-bound inhibitor Kelch-like ECH-associated protein 1 (Keap1). Keap1 subjects Nrf2 to polyubiquitination and degradation via the proteasome. During redox imbalance, or if the cell is exposed to electrophilic small molecules, key cysteine residues on Keap1 are oxidized causing a conformational change that releases Nrf2. Under these conditions, Nrf2 is able to translocate to the nucleus where it forms heterodimers with Maf proteins and binds ARE/EpRE. Nrf2 binding to ARE/EpRE results in transcription of cytoprotective enzymes such as catalase, glutathione S-transferase, NAD(P)H quinone oxidoreductase 1, SOD 1, heme oxygenase 1, and many more.

#### 5.1.1. Sulforaphane

Sulforaphane (SFN) is an isothiocyanate metabolite found in cruciferous vegetables, such as broccoli, and is one of the most widely studied electrophilic Nrf2 inducers. The anticarcinogenic properties of broccoli were attributed to SFN when it was first identified as a phase II metabolic enzyme inducer in 1992 [147]. After the characterization of the Nrf2/Keap1 pathway, SFN was identified as a Nrf2 inducer further describing its potential in anticancer therapy [148]. Since then, oral administration of SFN has been shown to improve behavioral symptoms of young men with autism spectrum disorder [149] and is being further evaluated for this condition (NCT02561481, NCT02879110, and NCT02909959). SFN has also been shown to improve fasting glucose in obese patients with dysregulated type 2 diabetes [150] and is currently in a phase II clinical trial (NCT02801448). As a redox-based therapeutic, SFN is making headway in animal models of atherosclerosis. Angioplasty with stent placement is the most common surgical intervention for treating atherosclerosis or a stenotic vessel. A major complication following angioplasty is vessel reocclusion or restenosis. Currently, restenosis is best inhibited by chemotherapy-eluting stents, yet they create a favorable local environment for thrombosis [151]. Yoo et al. [152] showed that local application of SFN at the site of arterial injury inhibits restenosis in an angioplasty-injury rat model. This was attributed to the Nrf2-activating properties of SFN, which likely inhibits the phenotypic change in vascular smooth muscles that drives restenosis. Multiple other groups have since successfully inhibited restenosis using SFN in similar animal models [153, 154]. One of us has been studying local delivery of other electrophiles such as cinnamic aldehyde as a therapy to inhibit restenosis. Development of a delivery vehicle that specifically targets the site of an atherosclerotic plaque could provide a novel antioxidant approach for treating vasculopathies and improving surgical outcomes and is the current focus of our lab.

#### 5.1.2. Dimethyl Fumarate

Dimethyl fumarate (DMF) is an esterified fumaric acid that can inhibit NF-*κ*B activation [155]. DMF effectively increases relapsing-remitting multiple sclerosis patient quality of life when administered orally as a delayed-release conformation, termed BG00012 [156] (NCT00420212). DMF interacts with GSH under specific conditions in the bloodstream which limits this absorption route and bioavailability, justifying why recent studies mostly use limited and/or low doses of DMF [157]. New formulations of DMF or newly synthesized derivatives could improve the pharmacokinetics of this compound and provide useful new approaches.

#### 5.1.3. Nitro-Oleic Acid

Nitro-oleic acid is one of the many nitrated fatty acids naturally present in human and animal tissues. Nitrated fatty acids result from ^•^NO-derived species reacting with unsaturated fatty acids resulting in ^•^NO_2_ addition at a double bond [158]. Nitro-oleic acid has been shown to have cytoprotective properties through Nrf2 and PPAR*γ* activation and NF-*κ*B inhibition [158]. CXA-10 is an orally administered 10-nitro-oleic-acid compound developed by Complexa Inc. CXA-10 has been trialed in five phase I clinical trials related to kidney injury as of 2017 (NCT02313064, NCT02127190, NCT02248051, NCT02460146, and NCT02547402) with differentiated safety in over 100 subjects reported as well as activation of target genes and inhibition of disease-related fibrotic and inflammatory biomarkers. Notably, the metabolic profile of patients in NCT02127190 has recently been characterized [159]. CXA-10 is set to enter phase II trials in 2018 for the treatment of pulmonary arterial hypertension and focal segmental glomerulosclerosis, two life-threatening diseases with a severely poor prognosis. These and future trials using nitrated lipids may yield effective redox-based therapies.

#### 5.1.4. Synthetic Electrophiles

Recently, there has been a surge in development of ARE-/EpRE-inducing molecules. Such molecules include N-iodoacetyl-N-biotinylhexylenediamine (IAB) which modifies Keap1 [160] but so far has a minimum effect on Nrf2 activation due to cytotoxicity and engagement with intracellular proteins [161]. Dai et al. [162] recently showed that several new formulations of Pyrazino[2,1-*a*]isoquinolin analogues, compounds originally designed for their antifungal properties [163], have potential as inducers of the Nrf2 pathway. Future studies using novel formulations may yield exciting therapeutics in the upcoming years. Moreover, testing new synthetic electrophilic compounds using novel platforms for targeted delivery could offer significant advances in redox medicine.

### 5.2. Nitric Oxide and Related Compounds

^•^NO is a small nitrogen-centered free radical gas that can diffuse several cell diameters from its production site. As mentioned above, it is synthesized by nitric oxide synthases (NOS). It serves as a neurotransmitter involved in memory and learning, and as a signaling molecule controlling vascular tone, cell proliferation, and cell survival. During inflammation an inducible form of NOS (iNOS) produces large quantities of ^•^NO involved in host protection against pathogens. Because of its role in vascular homeostasis, its potential therapeutic role in vascular disease has been extensively studied and is reviewed elsewhere [164]. Direct inhalation of ^•^NO is the simplest way to deliver it. Inhaled ^•^NO has been clinically used to treat pulmonary hypertension and acute respiratory distress syndrome. However, a recent systematic review analyzing 14 different trials shows that even though inhaled nitric oxide improves oxygenation, there is no reduction in mortality [165]. On the other hand, inhalational ^•^NO appears to be beneficial for respiratory failure in terms of infants after meta-analysis of 16 studies [166]. ^•^NO reduced mortality and/or use of extracorporeal membrane oxygenation. Moreover, inhalational ^•^NO reduces development of chronic lung disease and use of extracorporeal membrane oxygenation in newborns with persistent pulmonary hypertension [167]. Similarly, Miller et al. [168] conducted a randomized study in infants with pulmonary hypertension after congenital heart surgery. They found that inhalation of ^•^NO lessens the risk of pulmonary hypertensive crises and shortens postoperative recovery [168].

Since ^•^NO is highly reactive, inhalational ^•^NO is mostly a local intervention to the airways. When it comes to systemic administration, different strategies have been used to increase ^•^NO: administration of its precursors L-arginine, use of ^•^NO donors, or use of other ^•^NO bioactive compounds such as nitrates, nitrite, or S-nitrosothiols. A systematic review in 2017 that included 5 completed clinical trials assessing the use of glyceryl trinitrate in patients with stroke concluded that there is no evidence to recommend use of this drug in stroke [169]. The amount of preclinical data suggesting a beneficial effect of increasing ^•^NO for the treatment of vascular disease has not led to a successful clinical translation. Even though there are several attempts to increase ^•^NO via systemic administration of L-arginine or nitric oxide donors, they have failed to improve vascular outcomes (reviewed in [164] and [170]). A large prospective multicenter, randomized trial studying the effect of ^•^NO donors on restenosis after balloon angioplasty showed a modest improvement on angiographic restenosis but no differences in clinical outcomes [171]. These negative results highlight the issue of attempting to treat a localized ^•^NO deficiency at sites of arterial disease via a systemic approach. Hence, numerous attempts to deliver bioactive ^•^NO locally aim at overcoming the problems with systemic delivery. Endolumenal delivery of ^•^NO via a permeable balloon catheter inhibits restenosis in the rat carotid balloon injury model [172]. We have shown that an S-nitrosated nanofiber targeted to sites of arterial injury significantly inhibits restenosis in the rat balloon carotid injury model [173].

Further evidence that targeted or localized approaches result in successful clinical application of ^•^NO-based therapies is provided from fields that lend themselves to formulations for local delivery. One such field is dermatology, and the anti-inflammatory and antimicrobial properties of ^•^NO show promise to treat acne vulgaris [174]. Results of a phase II clinical trial (NCT01844752) show that a topical formulation of ^•^NO decreases inflammatory and noninflammatory lesions in subjects with acne vulgaris [175].

### 5.3. Flavonoids

Flavonoids are a group of naturally occurring compounds found in many fruits and vegetables as well as beverages such as tea and wine. The flavonoid active compounds are secondary metabolites that have been proven beneficiary in protecting against atherosclerotic disease progression [176]. Flavonoids were initially identified for their direct scavenging properties but recent findings point out that the indirect antioxidant effects are of greater biological relevance and better support the use of these compounds as therapeutics [177]. Accordingly, flavonoids have been ascribed to anti-inflammatory, anticancer, and antithrombotic properties with dose-dependent effects [178].

#### 5.3.1. Curcumin

*Curcuma longa*, turmeric, is the yellow-pigmented spice commonly used in curries that is comprised of naturally occurring phenolic compounds called curcuminoids. Curcumin is the most abundant of the curcuminoids and is classified as a linear diarylheptanoid with multiple functional groups. In mice, curcumin has been shown to induce Nrf2 nuclear translocation and induction of ARE/EpRE signaling [179]. Curcumin has been shown to inhibit gastric tumor growth by disrupting cellular bioenergetics [180] and to reduce the atherogenic and dyslipidemic panel in patients with chronic disease [181, 182]. Multiple studies show adjunct supplementation of curcumin with other active ingredients induces redox homeostasis in metabolic syndrome [183]. Of note, curcumin has been extensively pursued as a therapeutic with over $150 million in federal funds linked to biomedical studies, yet no conclusive therapeutic effects in humans have been determined to date [184]. Nelson et al. [184] recently published an extensive review describing some considerations to explain the pitfalls of using curcumin. In general, the poor absorption of curcumin and limited abundance of the active compound may hinder its therapeutic potential as a systemic agent. Furthermore, they classify curcumin as a pan-assay interference compound which may show activity in an assay by interfering with the readout rather than by the specific compound/target interaction the assay aims to assess. Nevertheless, because of its lipophilic characteristics, curcumin could still be considered a good agent for synergic localized or targeted therapy if better formulations arise. Indeed, nanoparticle-encapsulated curcumin has been shown to have antimicrobial properties and to promote wound healing in an *in vivo* murine wound model [185].

#### 5.3.2. Quercetin

Quercetin is a polyphenolic, pigmented flavonoid found in capers and onions that acts as an ROS scavenger. Quercetin has been proposed to have antioxidant effects in inducible vascular models of disease [186, 187], while also exhibiting anti-inflammatory and antineoplastic properties in several other models [188–190]. Unfortunately, most flavonoids are not readily absorbed by the human intestine which severely limits the bioavailability and overall therapeutic potential of quercetin [191]. It has failed to exhibit the anticipated preventative effects on serum phospholipids in healthy subjects as a supplement over an 8-week period [192]. Also, no significant changes in endothelial function or ^•^NO production were reported after 50–400 mg doses of water-dissolved quercetin extract were given to healthy subjects [193] (ACTRN12615001338550). It has been reported that a food matrix, as in onion powder rather than extract, significantly increases the bioavailability of quercetin in animals and humans [194]. Burak et al. [195] recently showed that the use of concomitant onion skin extract, instead of quercetin dehydrate alone, significantly increased the oral bioavailability of quercetin in healthy subjects. Extracting quercetin during food processing may be the factor limiting bioavailability of this flavonoid. As such, combinatorial therapy with other antioxidants, as has recently been trialed [196], may enhance quercetin delivery and overall benefit.

#### 5.3.3. Resveratrol

Resveratrol is a natural phenol found in berries. It is the most studied flavonoid in scientific research and has the potential to induce Sirtuin-1 activity [197] and modulate Nrf2 [198] and NF-*κ*B [199]. *In vitro* studies indicate anti-inflammatory effects from resveratrol in multiple cell lines [200, 201]. In human keratinocytes, it has been shown to reduce H_2_O_2_-induced production of ROS [202]. It also has proven preventive antioxidant therapeutic effects in multiple animal injury models [203–205]. In chickens, resveratrol induces redox balance via heat shock protein regulation in immuno-competent tissues [206]. In clinical studies, oral supplementation in adult smokers significantly decreased acute phase markers and triglycerides but did not alter chronic disease markers, weight, and blood pressure [207]. Topical application of resveratrol and vitamin E increased skin pigmentation and elasticity by altering heme oxygenase 1 and other collagen-specific targeting genes [208]. Orally administered resveratrol reduced cardiovascular disease risk in hypercholesterolemia patients and also increased serum vitamin E levels [209]. Apostolidou et al. [209] highlight the synergistic properties of resveratrol and vitamin E in decreasing cardiovascular disease risk. This illustrates the great potential that flavonoids, specifically resveratrol, might have as a joint therapy with other antioxidant molecules.

### 5.4. Thiol Molecules

#### 5.4.1. Glutathione

Glutathione (GSH) is a nonprotein, tripeptide thiol and one of the most abundant antioxidants in the body. In its reduced form, GSH is integral for cellular metabolism and maintaining cellular redox homeostasis. Work done by Mischley et al. [210] suggested that intranasal delivery may be able to augment GSH levels within the nervous system of patients with Parkinson's disease. In a follow-up phase II trial, however, there was no significant improvement in motor function from intranasal GSH delivery compared to the placebo [211]. In 2015, Schmitt et al. [212] showed that sublingual GSH was absorbed more effectively than oral supplementation by bypassing hepatic metabolism, potentially revealing a compelling new delivery approach. Several clinical trials assessing the benefit of oral GSH delivery are currently underway for growth improvement in children with cystic fibrosis (NCT03020719) and overall health of adults with a history of malnutrition (NCT03166371). Future work will dictate whether the therapeutic benefits of GSH can be through isolated administration or will continue to rely on intracellular upregulation by exogenous agents.

#### 5.4.2. N-Acetylcysteine

N-acetylcysteine (NAC) is a prodrug thiol and precursor for cysteine and GSH. NAC treatment has been promising in *in vitro* [213] and *in vivo* [214] models but has fallen short in clinical trials similarly to most other antioxidants. NAC was ineffective in randomized clinical studies of arterial disease [215] and in attenuating nephrotoxicity [216]. Kazemi et al. [217] found no association from a high dose of prophylactic NAC given orally before and after surgery in preventing postoperative atrial fibrillation. A clinical trial evaluating NAC administered with amiodarone, which helps regulate heart rhythm, in preventing atrial fibrillation after thoracic surgery is currently in phase III (NCT02750319). Synergic NAC administration has been promising in treating infection [218] and reflux disease [219]. Trials using NAC with L-carnitine for polycystic ovary syndrome (NCT03164421) and with the drug minocycline for the treatment of bipolar depression (NCT02719392) are currently underway. As of 2017, there are over 70 other ongoing clinical trials using NAC as a sole therapeutic or together with other antioxidant molecules or drugs which will broaden our understanding of this unique prodrug.

### 5.5. Other Scavenging Molecules

#### 5.5.1. Coenzyme Q10 and MitoQ10

Coenzyme Q10 (CoQ10) is a lipid-soluble ubiquinol antioxidant with high functionality in the Golgi apparatus and endoplasmic reticulum [220]. CoQ10 has been shown to reduce oxidative stress by modulating UBIAD1 and eNOS enzymatic activity in cardiovascular tissues [221]. A research group from Linköping University in Sweden has published multiple papers using oral administration of CoQ10 in clinical studies. The results show a direct effect on serum selenium levels which corresponds with decreased cardiovascular mortality rate in elderly patients [222]. CoQ10 in joint administration with selenium modified over 100 different microRNA expression levels in healthy elderly patients [223]. Lastly, the joint administration with selenium for 4 years increased insulin-like growth factor-1, which has implications of anti-inflammatory and antioxidative effects, further explaining the observed reduction in cardiovascular mortality in these trials [224]. Yet, a 300 mg oral dose of CoQ10 12 hours postprocedure did not reduce periprocedural myocardial injury in patients with elective percutaneous coronary interventions but did decrease C-reactive protein [225]. MitoQ10 is a 3rd generation derivate of CoQ10 that has an added positive charge with direct targeting for the mitochondria. Even though this approach does not target a specific organ or tissue, it directs the redox intervention to a specific organelle. MitoQ10 administered in drinking water jointly with Losartan prevented hypertensive disease and consequent rat myocardial hypertrophy [226]. In a clinical trial, orally administered MitoQ10 showed no therapeutic benefit as a Parkinson's disease-modifying therapy [227]. MitoQ10 is currently in clinical trials for improving physiological function of middle-aged and older adults (NCT02597023), attenuating chronic kidney disease (NCT02364648), and reducing fatigue experienced by patients with multiple sclerosis (NCT03166800). The benefits of these two therapeutics appear yet to come, especially for MitoQ10 as it advances into promising clinical trials.

#### 5.5.2. Ebselen

Ebselen is a synthetic selenoorganic molecule with antioxidant, anti-inflammatory, antimicrobial, and antifungal properties. This molecule is capable of catalytically scavenging hydrogen peroxide and organic hydroperoxides, at the expense of cellular thiols, thus mimicking glutathione peroxidase [228]. Additionally, ebselen can react with ONOO^−^ (k = 10^6^ M^−1^ s^−1^) providing another possible mechanism of redox modulation [229]. Moreover, the ability of ebselen to react with cellular thiols makes ebselen a weak electrophile capable of activating Nrf2 [230]. SPI-1005 is an oral formulation of ebselen developed by Sound Pharmaceuticals Inc. that has undergone phase I trials for hearing loss (NCT01452607) and Meniere's disease (NCT02603081) and phase II trials for preventing noise-induced temporary auditory threshold shifts (NCT01444846) [231]. Currently, ebselen is in phase II trials to prevent ototoxicity (NCT02819856), as well as noise-induced (NCT02779192) and chemotherapy-induced (NCT01451853) hearing loss. Preclinical evidence suggested that ebselen improves insulin secretion and pancreatic islet viability in murine models of diabetes [232, 233]; however, ebselen failed to improve glycemia and vascular function in a small clinical trial with diabetic patients [234]. Importantly, this trial measured redox markers and showed that the ebselen dose and regimen used did not reduce any systemic markers of oxidative stress, suggesting that redox regulation was not achieved. The failure of this trial should not be taken to imply that redox imbalance is not at the core of the disease. Rather, as the redox state was not altered, this suggests that a wrong dose, improper administration route, or perhaps even the wrong redox-active molecule was used. Ongoing work with different formulations of selenoorganic molecules [235] may yield new and interesting therapeutics.

#### 5.5.3. Edaravone

Edaravone is a synthetic reactive species scavenging antioxidant previously named MCI-186. Edaravone has been reported to specifically quench hydroxyl radicals, thus preventing lipid peroxidation, and has been used for the treatment of ischemia-reperfusion injury in Japan for more than a decade [236]. Edaravone was rebranded as Radicava in 2017 after its approval by the U.S. Food and Drug Administration (FDA) for the treatment of amyotrophic lateral sclerosis (ALS) [237], a rare neurodegenerative disease that effects voluntary muscle movement. This designation was made after the conclusion of the final phase III trial in October of 2017 (NCT01492686). Radicava treatment significantly reduced the rate of disability progression experienced by patients with ALS. The FDA approval of an antioxidant molecule shows promise that identifying the proper disease and patient population for a given molecule can result in a successful drug campaign and, more importantly, in an effective redox-based therapy.

## 6. Conclusion

A causal relationship linking redox dysfunction with disease would predict successful translation to the clinic of therapies aimed at restoring redox homeostasis. However, the complexity of cellular redox reactions continues to thwart the utility of antioxidants as effective therapeutics in disease prevention and treatment. The first trials aimed at restoring redox homeostasis utilized antioxidants for their direct scavenging and redox cycling properties but were largely unsuccessful. Current evidence suggests that using molecules to upregulate endogenous antioxidant enzyme expression can have useful therapeutic benefits. Likewise, inhibition of ROS/RNS producing enzymes or inhibiting pathways that promote excess ROS/RNS generation could ultimately yield a greater efficacy than attempting to scavenge these species directly. Early failures of redox-active molecules in clinical trials may reflect the wrong choice of therapeutic, the wrong dose, or wrong selection of a patient population that would benefit the most from the intervention. Not accounting for these possibilities might lead to the conclusion that redox dysfunction is not part of the disease etiology. Importantly, few studies actually assess the effect of the redox intervention on redox biomarkers. If an antioxidant fails to improve primary outcomes, it is important to know if it actually affects redox markers. If the intervention does not improve redox status, then it is likely that the selection of drug, dose, or route of administration was not ideal. This issue does not have a clear solution since there is not a consensus as to what biomarkers to use when assessing redox function *in vivo*. Having some evidence that the tested drug actually improves redox homeostasis, however, allows for the identification of a subset of patients that are more likely to benefit from the redox intervention. For example, the use of tirapazamine, a reductively activated drug to potentiate chemotherapy in patients with squamous cell carcinoma, showed a promising significant effect in patients with hypoxic tumors [238]. However, in a large phase III trial without selection for hypoxia, addition of tirapazamine to chemoradiotherapy showed no improvement in outcomes [239]. This highlights the need to identify the patient population that is most likely to benefit from the intervention. Failure to identify this subpopulation could dilute the patient pool potentially masking beneficial effects. Similarly, the example shows the importance of measuring the specific conditions related to the expected mechanism of action. Current studies address several of these issues. Importantly, many successful clinical trials have exploited local delivery or a targeted effect to specific cells. The realization that local and targeted drug delivery could directly address the problem of systemic effect of redox therapies opens a new and exciting avenue for translational redox research. As the drug delivery field develops new and better ways to precisely target therapeutic payloads to tissue sites, we can specifically address local redox dysfunction.

## Figures and Tables

**Table 1:** Preclinical and clinical studies using vitamin C.

Vitamin C		
Route	Results	Reference
*Preclinical studies*		
*In vitro*	Pancreatic cancer cell H_2_O_2_ production increases susceptibility to therapeutic effect of vitamin C	[20]
I.P.	Maintaining a 20 mM plasma concentration of vitamin C is optimal to obtain a therapeutic effect from vitamin C in an induced-tumor mouse model	[20]
*In vitro*	Vitamin C increases intracellular iron uptake in HUVECs as well as in multiple human cancer cell lines	[19]
*In vitro*	Vitamin C selectively sensitizes non-small-cell lung cancer and glioblastoma cells to radiation and chemotherapy, while increasing labile iron and H_2_O_2_ levels	[18]

*Clinical studies*		
1.25 g (oral) 50 g (I.V.)	Orally administered vitamin C unable to maintain therapeutic plasma concentration	[21]
15–125 g I.V.	High-dose infusion of vitamin C with gemcitabine in stage IV pancreatic cancer patients could achieve stable plasma levels of 20 mM while increasing the mean survival time	[23]
75 g I.V.	Vitamin C infusions were well tolerated and increased average progression-free survival in glioblastoma patients and control rate of disease in non-small-cell lung cancer patients	[18]

I.P.: intraperitoneal; I.V.: intravenous.

**Table 2:** Preclinical and clinical studies using vitamin E.

Vitamin E		
Route	Results	Reference
*Preclinical studies*		
*In vitro*	*α*-Tocopherol activates Nrf2 in human retinal pigment epithelial cell line ARPE-19, thus inducing transcription of phase II enzymes	[32]
Oral	16 weeks of 1500 IU vitamin E daily is able to rescue Nrf2 function in alveolar macrophages from human atopic asthmatics	[33]
*In vitro*	*α*-Tocopheryl succinate activates Nrf2 in PC3 prostate cancer cell line which inhibits NF-*κ*B nuclear translocation and neoplastic activity	[31]
Oral I.M.	Vitamin E significantly reduced atherosclerotic plaque progression in rabbits fed a high-cholesterol diet	[26]
Oral	Vitamin E significantly reduced atherosclerotic plaque progression in ApoE^−/−^ mice	[27]
Oral	Vitamin E deficiency disrupts grass carp growth and physiology while vitamin E supplementation is able to reverse the negative effects	[34]

*Clinical studies*		
400–800 IU Oral	Daily vitamin E supplementation showed substantial reduction in nonfatal myocardial infarction in patients with angiographically proven coronary atherosclerosis, yet no significant benefit on risk of cardiovascular death was observed	[38]
400 IU Oral	4–6 years of daily vitamin E supplementation showed no therapeutic benefit on cardiovascular events in high-risk patients 55 years of age or older	[39]
100–3200 IU Oral	16 weeks of at least 800 IU/day of vitamin E required to reduce the plasma F2-isoprostane concentration	[40]
400 IU Oral	Daily supplementation of vitamin E only therapeutic in type 2 diabetes with genotype for systemically elevated oxidative stress	[41]
400 IU Oral	Hemodialysis patients experienced reduced rate of plasma MDA level increase following 2 months of vitamin E supplementation.	[44]
400 mg Oral	Patients with Down syndrome had their abnormal superoxide dismutase and catalase activity as well as low levels of reduced glutathione returned to physiological levels following vitamin E supplementation	[45]
2000 IU Oral	Daily high dose of vitamin E slowed the functional decline of Alzheimer's disease patients	[46]
400 IU Oral	Eight weeks of daily vitamin E supplementation increased paraoxonase-1 enzyme activity but did not lower serum malondialdehyde levels in type 2 diabetic patients	[43]
300 mg Oral	Three months of daily vitamin E supplementation significantly reduced serum malondialdehyde levels in insulin-dependent type 2 diabetic patients	[42]
Ointment S.C.	Patients undergoing colorectal cancer surgery exposed to vitamin E at the surgical site experienced a reduced rate of surgical site infection and lowered inflammatory response	[47]

I.M.: intramuscular; S.C.: subcutaneous.

**Table 3:** Preclinical and clinical studies using alpha-lipoic acid.

Alpha-lipoic acid		
Route	Results	Reference
*Preclinical studies*		
Oral	ALA slowed the rate of plaque progression in a diet-induced rabbit atherosclerosis model	[56]
I.P.	ALA reduced lesion size in a diet-induced atherosclerotic mouse model. ALA also reduced vascular smooth muscle cell proliferation and migration *in vitro*	[59]
I.V.	Spleen weight/body weight ratio, levels of H_2_O_2_, lipid peroxidation, and levels of reduced glutathione returned to physiological levels in LPS-treated rats following ALA injection	[52]
Oral	ALA restored GSH, SOD, and catalase plasma concentrations to physiological levels in a pesticide-induced oxidative stress rat model	[54]
S.C.	ALA restored SOD and catalase concentrations to physiological levels in brain tissue of a phenylketonuria rat model	[55]
I.P.	Kidney mitochondrial function restored in LPS-treated rats following ALA exposure	[53]

*Clinical studies*		
600–1800 mg Oral	Five weeks of daily ALA supplementation alleviated the pain experienced by diabetics suffering from distal symmetric polyneuropathy.	[61]
600 mg Oral	Four years of daily ALA supplementation improved neuropathic conditions of diabetics suffering from polyneuropathy and the 600 mg dose was well tolerated for an extended period of time	[63]
600 mg Oral	20 weeks of daily ALA supplementation alleviated the pain experienced by diabetics suffering from polyneuropathy	[62]

I.P.: intraperitoneal; I.V.: intravenous; S.C.: subcutaneous.

**Table 4:** Clinical studies using vitamin A.

Vitamin A		
Route	Results	Reference
*Clinical studies*		
24 mg Oral	12 weeks of daily oral vitamin A reduced UV-induced skin lesions	[73]
0.4% lotion Topical	Topical vitamin A application three times a week for 24 weeks reduced aging-related wrinkles	[74]
0.2% lotion Topical	Topical vitamin A protects skin from IR-generated reactive species	[72]
20 mg Oral 0.05% lotion Topical	Topical vitamin A more effective in treating photoaging than orally administered	[77]

**Table 5:** Preclinical and clinical studies using vitamin B_12_.

Vitamin B_12_ (cobalamin)		
Route	Results	Reference
*Preclinical studies*		
*In vitro*	Pulse radiolysis determined that the rate constant between Cob(II)alamin and superoxide (O_2_^•−^) is 6.8 × 10^8^ M^−1^ s^−1^, which is within an order of magnitude of cytosolic and mitochondrial superoxide dismutase (SOD)	[79]
*In vitro*	Cob(II)alamin reacts with O_2_^•−^ at a rate similar to that of the SOD	[80]
*In vitro*	Cyanocobalamin treatment inhibited O_2_^•−^-induced damage in human aortic endothelial cells by scavenging intracellular O_2_^•−^	[83]

*Clinical studies*		
0.5 mg Oral	Daily supplementation with folic acid, vitamin B_6_, and vitamin B_12_ for one year significantly reduced the carotid intima-media thickness, a marker of atherosclerosis, in at-risk patients	[86]
0.4 mg Oral	Six months of daily folic acid and vitamin B_12_ supplementation improved coronary endothelial function in patients at risk for coronary artery disease	[87]
0.4 mg Oral	Two years of daily supplementation with folic acid and vitamin B_12_ significantly increased coronary blood flow in patients with stable coronary artery disease	[88]
25 or 0.5 mg I.M.	Daily ultrahigh-dose injections (25 mg) of methylcobalamin improved the compound muscle action potentials of patients with amyotrophic lateral sclerosis	[89]
Eyedrops	Eyedrops containing 0.15% hyaluronic acid and vitamin B_12_ reduced oxidative stress markers in the conjunctiva (mucous membrane covering eye) and improved the overall symptoms of patients with chronic dry eye	[90]

I.M.: intramuscular.
